# Effect of 6S refined individualized nursing management in the perioperative period of Parkinson’s disease patients undergoing deep brain stimulation

**DOI:** 10.3389/fneur.2026.1671449

**Published:** 2026-06-03

**Authors:** Yunhui Luo, Fang Guo, Jing Bai, Xuan Li, Jinghua Bei, Yujing Xing, Feng Zhang

**Affiliations:** 1 Disinfection Supply Center, First Hospital of Hebei Medical University, Shijiazhuang, Hebei, China; 2 Department of Ophthalmology, First Hospital of Hebei Medical University, Shijiazhuang, Hebei, China; 3 Department of Neurosurgery, First Hospital of Hebei Medical University, Shijiazhuang, Hebei, China

**Keywords:** 6S refined individualized nursing, daily living ability, deep brain stimulation, Parkinson’s disease, perioperative

## Abstract

**Aim:**

This study aimed to investigate whether Sort, Set in order, Shine, Standardize, Sustain, and Safety (6S) refined individualized nursing management improves perioperative outcomes in patients with Parkinson’s disease (PD) undergoing deep brain stimulation (DBS).

**Methods:**

This single-center retrospective cohort study included 96 patients with PD who underwent DBS in our hospital from January 2022 to June 2025. According to the perioperative nursing model implemented during the study period, patients were allocated to either a control group (CG) receiving routine perioperative nursing (*n* = 48) or a study group (SG) receiving 6S refined individualized nursing management in addition to routine care (*n* = 48). We compared the operation time, time to first ambulation, postoperative length of stay (LOS), anxiety and depression [self-rating Anxiety Scale / Self-rating Depression Scale (SAS/SDS)], pain [visual analog scale (VAS)], balance [Tinetti Balance and Gait Assessment (POMA)], activities of daily living (Barthel index), complication rates, and nursing satisfaction between groups.

**Results:**

Compared to the control group, the study group had a shorter time to first ambulation (13.41 ± 1.51 vs. 14.89 ± 2.74 h) and a shorter postoperative LOS (10.34 ± 0.71 vs. 11.22 ± 0.95 days). After nursing, SAS scores were lower in the study group compared to the control group (21.52 ± 2.15 vs. 30.25 ± 3.06), as well as lower SDS scores (20.25 ± 2.04 vs. 30.26 ± 3.05). Postoperative VAS pain grades were consistently lower in the study group, for example, at 24 h after surgery (1.21 ± 0.12 vs. 2.05 ± 0.21). POMA scores and Barthel index values at discharge were higher in the study group compared to the control group (17.42 ± 1.75 vs. 15.26 ± 1.53 and 63.58 ± 6.45 vs. 60.25 ± 6.05, respectively). The incidence of nursing-recorded complications was lower in the study group than in the control group (2.1% vs. 14.6%), and overall nursing satisfaction was higher as well (97.9% vs. 83.3%).

**Conclusion:**

In this single-center retrospective cohort, 6S refined individualized nursing management was associated with faster postoperative recovery, reduced anxiety and depression, improved balance ability and activities of daily living, and fewer nursing-recorded perioperative complications in PD patients undergoing DBS. These findings should be regarded supportive but preliminary, and they require confirmation through larger prospective studies.

## Introduction

Parkinson’s disease (PD) is a common chronic neurodegenerative movement disorder characterized by bradykinesia, rigidity, resting tremor, and postural instability, and its prevalence increases markedly with age (1). In China, the incidence of PD among individuals aged >65 years is approximately 1.7%, and the prevalence increases with age; men are affected slightly more often than women (2). The clinical manifestations mainly contain static tremor, bradykinesia, and myotonia, as well as postural gait disorders, and may be accompanied by non-motor symptoms, including depression, constipation, and sleep disorders (3). Due to the lack of a clear etiology to date, it is believed that dopamine deficiency caused by the degeneration of substantia nigra cells is the key cause of this disease (4). Although levodopa is still the main treatment, the efficacy in some patients gradually decreases with the prolongation of the course of the disease, and increasing the dose of the drug will bring many intolerable adverse reactions (5). With the continuous improvement in medical standards, deep brain stimulation (DBS) has been gradually applied to patients with PD; that is, electrodes are implanted in specific nerve nuclei in the brain to release high-frequency electrical stimulation and repress the electrical impulse of neurons that are overexcited due to the reduction of dopaminergic neurons, reduce the overexcited state, and thus alleviate PD symptoms (6). At the same time, the operation may also cause complications such as electrode dislocation, electrode displacement, electrode rupture, puncture path bleeding, intracranial infection, and permanent nervous system damage (7). Therefore, in clinical practice, standard, meticulous, and skilled nursing cooperation is considered important for supporting perioperative safety in DBS and reducing the risk of complications, including infection (8).

6S management originated from the 5S model, which was originally developed in Japanese production enterprises and later introduced into healthcare (9). In Japanese, the five classic components are seiri (sort: remove unnecessary items from the workplace), seiton (set in order: organize and place necessary items so they are easy to find and use), seiso (shine: clean and maintain the work environment and equipment), seiketsu (standardize: maintain the highest standards of order and cleanliness), and shitsuke (sustain: cultivate discipline so that these practices are maintained over the long term). In many clinical nursing applications, a sixth “S” has been added-security-to emphasize patient and staff safety and risk prevention as an integral part of 6S-based nursing management (10). By optimizing the layout of the nursing unit, standardizing processes, and enhancing environmental safety, 6S management aims to create an orderly, clean, and efficient care environment and to improve the quality and safety of nursing services.

Herein, we aimed to explore the impact of 6S refined individualized nursing management during the perioperative period for PD patients undergoing DBS.

## Data and methods

### General data

This study retrospectively analyzed the clinical data of 96 patients with PD who underwent DBS in our hospital from January 2022 to June 2025, and the study protocol was reviewed and approved by the First Clinical Research Ethics Committee of Hebei Medical University (approval ID: [2023] Research Review No. (S00726); approval date: 28 June 2023). The inclusion criteria were as follows: (a) patients who met the diagnostic criteria for PD; (b) patients with indications for DBS; (c) complete and authentic clinical data. The exclusion criteria were as follows: (a) patients with malignant tumors and cardiovascular or cerebrovascular disease; (b) patients with other degenerative diseases of the nervous system; (c) patients with dysfunction or disorders of the heart, liver, kidney, or other major organs; and (d) patients with language, hearing, mental, or cognitive impairment. According to the nursing care implemented, patients were divided into a control group (CG, routine nursing, *n* = 48) and a study group (SG, 6S refined individualized nursing, *n* = 48). In this retrospective design, group allocation was primarily determined by the time period relative to the implementation of the 6S nursing model, with patients treated before implementation assigned to the control group and those treated after assigned to the study group. During the study period, the neurosurgical team, surgical techniques, and DBS-related equipment remained stable, and no major changes in perioperative medical management protocols were introduced. The CG included 28 males and 20 females, with a mean age of (64.23 ± 4.62) years (range, 56–81 years). The SG included 27 males and 21 females, with a mean age of (64.28 ± 4.65) years (range, 54–82 years). There were no significant differences in baseline characteristics between the two groups (*p* > 0.05).

## Methods

The CG adopted routine perioperative nursing according to the standard departmental protocol for DBS. Nurses performed basic preoperative assessment and general health education, monitored vital signs and neurological status, assisted with routine postoperative positioning and limb protection, implemented conventional pharmacological analgesia and general measures for prevention of pressure sores and falls (e.g., regular turning, skin care, and environmental safety checks), and provided general psychological support and discharge guidance. This protocol was consistent with our institutional standard-of-care for perioperative nursing management in DBS patients during the study period.

Based on the above routine care, the SG adopted 6S refined individualized nursing management. At environmental and organizational levels, the 6S principles (Sort, Set in order, Shine, Standardize, Sustain, and Safety) were applied to reorganize the ward environment and nursing workflows: DBS-related materials and devices were sorted, labeled, and stored in a standardized manner; medication, electrodes, and wound-care supplies were placed in clearly designated and visually managed locations; the cleanliness and safety of the ward were maintained through regular inspection; and standardized operating procedures, visual flowcharts, and documentation forms were developed for key perioperative nursing steps (preoperative preparation, intraoperative coordination, postoperative monitoring, early mobilization, and complication surveillance). At the patient level, a responsible nurse conducted a structured preoperative assessment (including age, disease duration and main symptoms, comorbidities, functional status, cognitive and emotional state, and family support) and formulated an individualized nursing plan specifying personalized goals and schedules for health education, early mobilization, pain management, fall and pressure sore prevention, and psychological support. The individualized plan was adjusted dynamically according to each patient’s daily recovery and feedback, and its implementation was supervised by the head nurse through regular ward rounds and review of nursing records. The primary components of routine nursing and 6S refined individualized nursing are summarized in Supplementary Table 1. To further clarify the intervention content and enhance reproducibility, Supplementary materials have been provided, including a domain-based comparison of routine perioperative nursing and 6S refined individualized nursing management (Supplementary Table 1) and a structured summary of the main perioperative and clinical outcomes (Supplementary Table 2).

### Observation indicators

Operation time, time to first ambulation, and postoperative length of stay (LOS) were compared between the two groups. Anxiety and depression were assessed using the Self-rating Anxiety Scale (SAS) and Self-rating Depression Scale (SDS) (11). SAS and SDS scores were measured within 24 h before surgery (“before nursing”) and again within 24 h before hospital discharge (“after nursing”). Pain was assessed using the visual analog scale (VAS) (12). For analysis, the raw VAS values (0–100 mm) were converted to a 0–4 pain grade (0 = no pain, 1 = mild pain, 2 = moderate pain, 3 = severe pain, 4 = very severe pain). VAS scores were recorded at 6, 12, 24, and 48 h after surgery. The Tinetti Balance and Gait Assessment (POMA) was used to assess the balance ability of patients (13), with total scores ranging from 0 to 28; higher scores indicate better balance and gait. POMA scores and Barthel index values were assessed within 24 h before surgery and within 24 h before discharge, in parallel with the SAS and SDS assessments. Activities of daily living were assessed using the Barthel index. The incidence of complications (electrode displacement, wound infection, and hypokalemia) was recorded and compared between the groups. Nursing satisfaction was evaluated using the hospital nursing evaluation scale (total score, 100): >90, very satisfied; 80–90, satisfied; <80, dissatisfied. Satisfaction was assessed once within 24 h before discharge. Total satisfaction was defined as the proportion of patients who were very satisfied or satisfied.

### Statistical analysis

SPSS 24.0 (IBM Corp., Armonk, NY, United States) was used for statistical analysis. Continuous variables are presented as mean ± standard deviation (x̄ ± s), and categorical variables are presented as *n* (%). The distribution of continuous variables in each group was examined using histograms and normal Q–Q plots, and the Shapiro–Wilk test was used to assess normality. Levene’s test was used to assess the homogeneity of variances. For the main continuous outcomes (operation time, time to first ambulation, postoperative LOS, SAS, SDS, VAS pain grades, POMA, and Barthel index), the distributions were approximately symmetric, and variances were comparable between groups; therefore, the assumptions for parametric tests were considered reasonably satisfied. Between-group comparisons of continuous outcomes were performed using independent-samples *t*-tests, and within-group pre–post comparisons (before vs. after nursing) for SAS, SDS, POMA, and the Barthel index were performed using paired *t*-tests. Between-group comparisons of categorical variables (complications and nursing satisfaction) were performed using χ^2^ tests. In addition to *p*-values, we calculated and reported mean differences with 95% confidence intervals (95% CI) and Cohen’s d effect sizes for continuous outcomes and absolute risk differences with 95% CI for categorical outcomes. Given the exploratory nature of this single-center study and the conceptual relatedness of the outcomes, no formal adjustment for multiple comparisons (e.g., Bonferroni or false discovery rate procedures) was applied. All tests were two-sided, and a *p*-value of <0.05 was considered statistically significant.

## Results

### Operation time, postoperative getting out of bed time and postoperative hospitalization time in two groups

**Figure 1:**
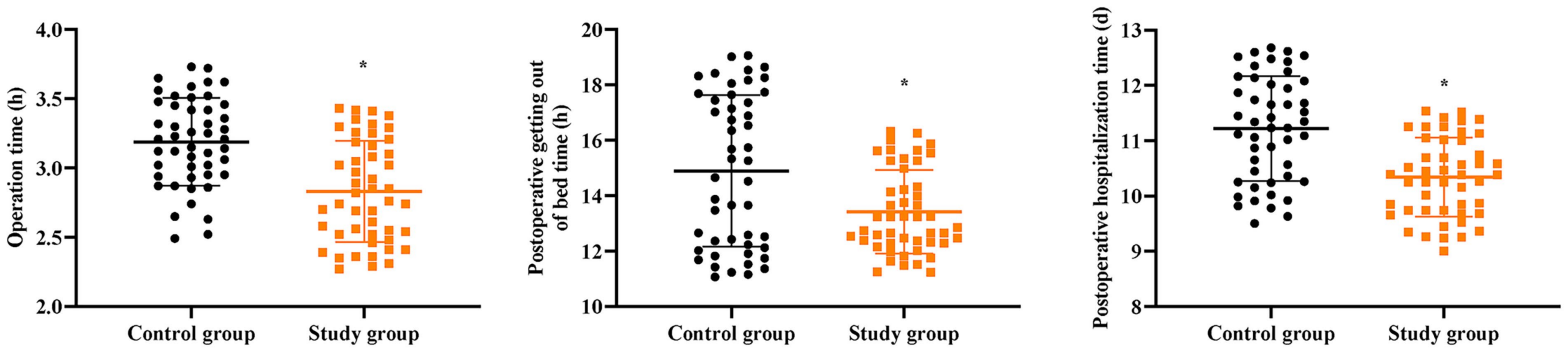
Operation time, postoperative getting out of bed time, and postoperative hospitalization time in two groups. ^*^ *p* < 0.05.

As shown in Figure 1 and Supplementary Table 2, the study group had a shorter operation time than the control group (2.83 ± 0.37 vs. 3.19 ± 0.32 h; mean difference −0.36 h, 95% CI -0.50 to −0.22; Cohen’s d = 1.05; *p* < 0.001), an earlier postoperative getting out of bed time (13.41 ± 1.51 vs. 14.89 ± 2.74 h; mean difference −1.48 h, 95% CI -2.37 to −0.58; Cohen’s d = 0.67; *p* = 0.002), and a shorter postoperative hospitalization time (10.34 ± 0.71 vs. 11.22 ± 0.95 d; mean difference −0.88 d, 95% CI -1.22 to −0.54; Cohen’s d = 1.05; *p* < 0.001).

### Anxiety and depression in patients in two groups

**Figure 2:**
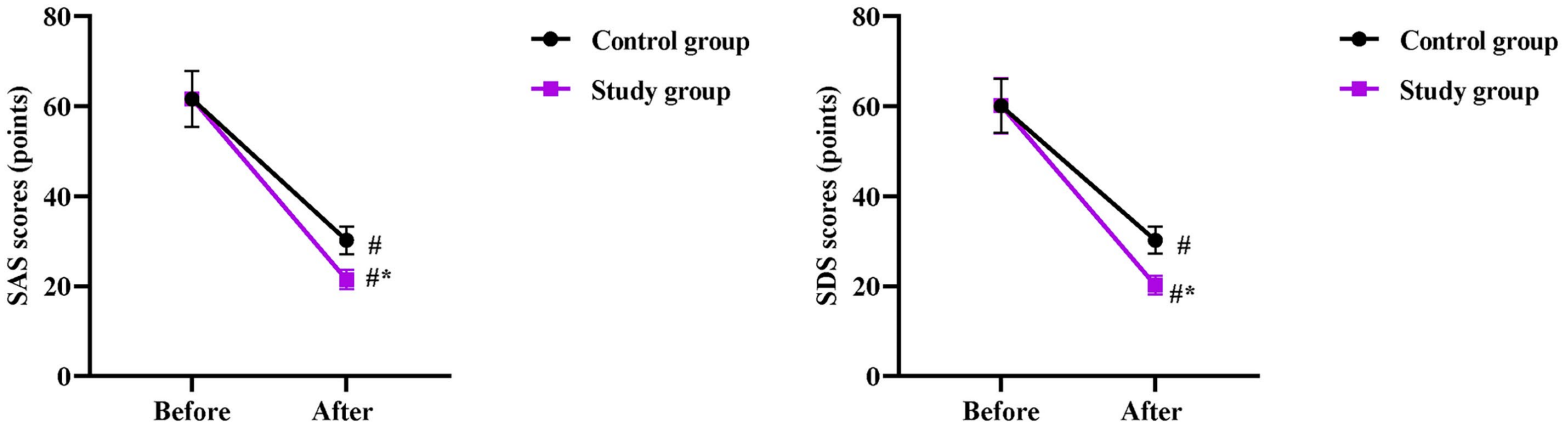
Anxiety and depression scores (SAS and SDS, mean ± SD) of patients in two groups before and after nursing. ^#^ *p* < 0.05 vs. before nursing within the same group; *p* < 0.05 vs. the control group after nursing.

Before nursing, there were no significant differences in SAS or SDS scores between the two groups (SAS: 61.65 ± 6.17 vs. 61.68 ± 6.21; mean difference 0.03, 95% CI -2.48 to 2.54; SDS: 60.12 ± 6.05 vs. 60.18 ± 6.13; mean difference 0.06, 95% CI -2.41 to 2.53; all *p* > 0.05). After nursing, SAS and SDS scores decreased in both groups, with markedly greater improvements in the study group: post-nursing SAS scores were 21.52 ± 2.15 vs. 30.25 ± 3.06 (mean difference −8.73, 95% CI -9.80 to −7.66; Cohen’s d = 3.30; *p* < 0.001), and SDS scores were 20.25 ± 2.04 vs. 30.26 ± 3.05 (mean difference −10.01, 95% CI -11.06 to −8.96; Cohen’s d = 3.86; *p* < 0.001) in the study and control groups (Figure 2; Supplementary Table 2).

### VAS scores in two groups

**Figure 3:**
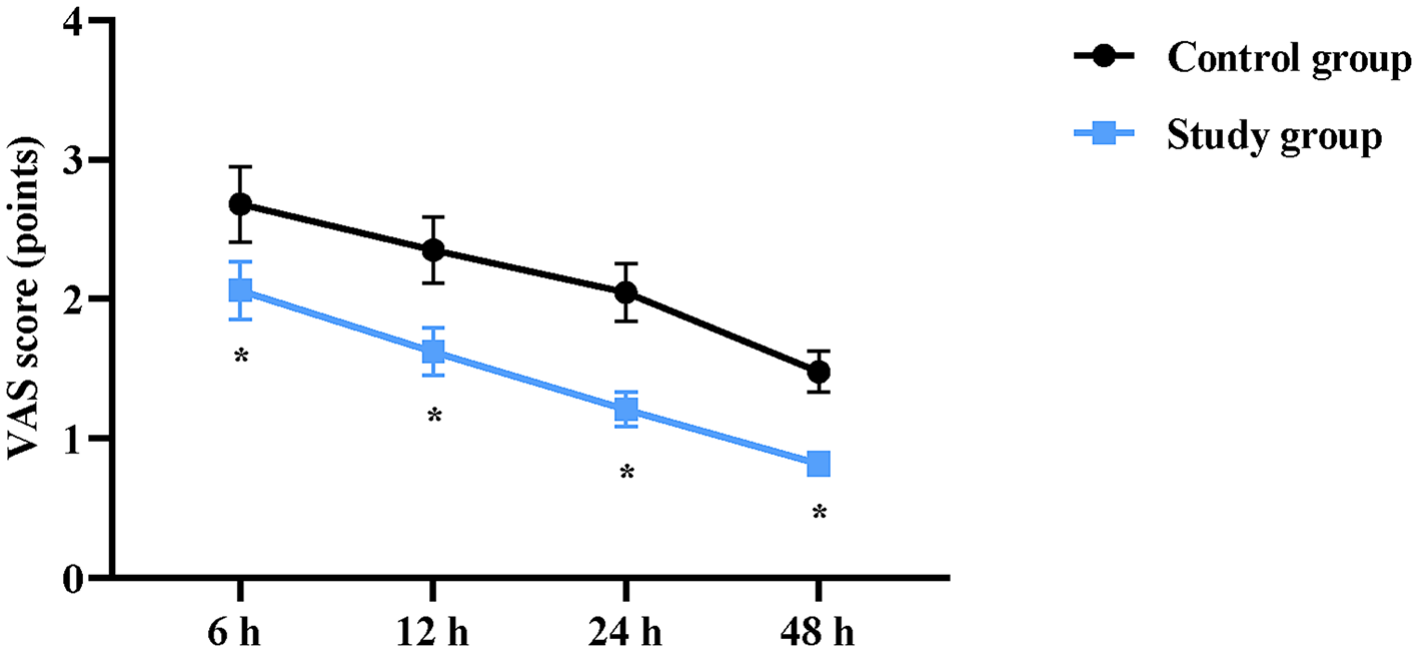
VAS pain scores (mean ± SD) in two groups at 6, 12, 24, and 48 h after surgery. Pain was initially assessed using a 100-mm VAS and then converted into a 0–4 pain grade (0–4 points). *p* < 0.05 vs. the control group at the same time point.

As shown in Figure 3 and Supplementary Table 2, postoperative pain intensity assessed by the VAS was consistently lower in the study group than in the control group at all time points after surgery: at 6 h (2.06 ± 0.21 vs. 2.68 ± 0.27; mean difference −0.62, 95% CI -0.72 to −0.52; Cohen’s d = 2.56; *p* < 0.001), 12 h (1.62 ± 0.17 vs. 2.35 ± 0.24; mean difference −0.73, 95% CI -0.81 to −0.65; Cohen’s d = 3.51; *p* < 0.001), 24 h (1.21 ± 0.12 vs. 2.05 ± 0.21; mean difference −0.84, 95% CI -0.91 to −0.77; Cohen’s d = 4.91; *p* < 0.001), and 48 h (0.82 ± 0.08 vs. 1.48 ± 0.15; mean difference −0.66, 95% CI -0.71 to −0.61; Cohen’s d = 5.49; *p* < 0.001).

### Balance ability of patients in two groups

**Figure 4:**
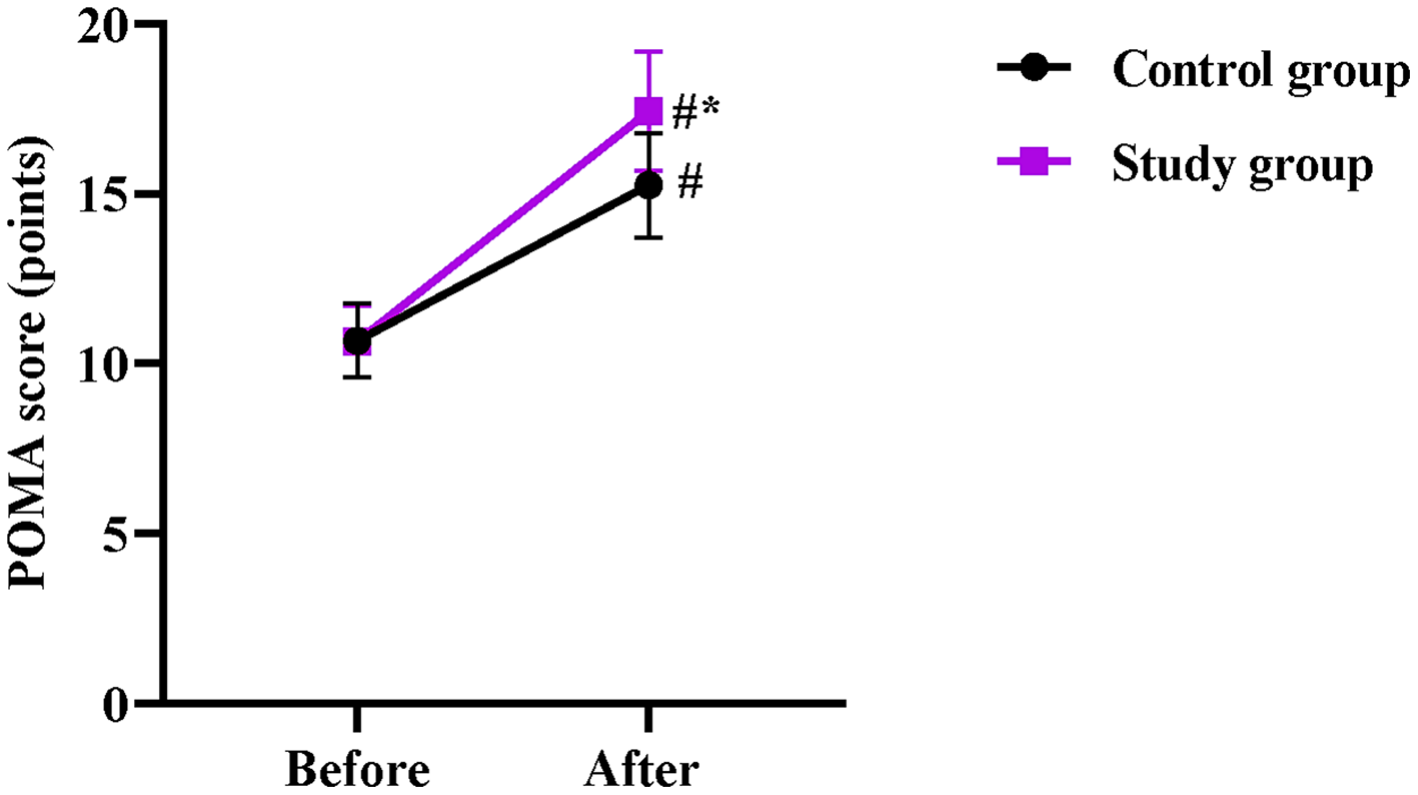
Balance ability scores (POMA, mean ± SD) of patients in two groups before and after nursing. ^#^ *p* < 0.05 vs. before nursing within the same group; *p* < 0.05 vs. the control group after nursing.

Before nursing, there was no significant difference in POMA scores between the two groups (10.68 ± 1.07 vs. 10.65 ± 1.05; mean difference −0.03, 95% CI -0.46 to 0.40; *p* > 0.05). After nursing, POMA scores increased in both groups, with significantly higher post-nursing scores in the study group (17.42 ± 1.75 vs. 15.26 ± 1.53; mean difference 2.16, 95% CI 1.49 to 2.83; Cohen’s d = 1.31; *p* < 0.001) (Figure 4; Supplementary Table 2).

### Barthel index in two groups

**Figure 5:**
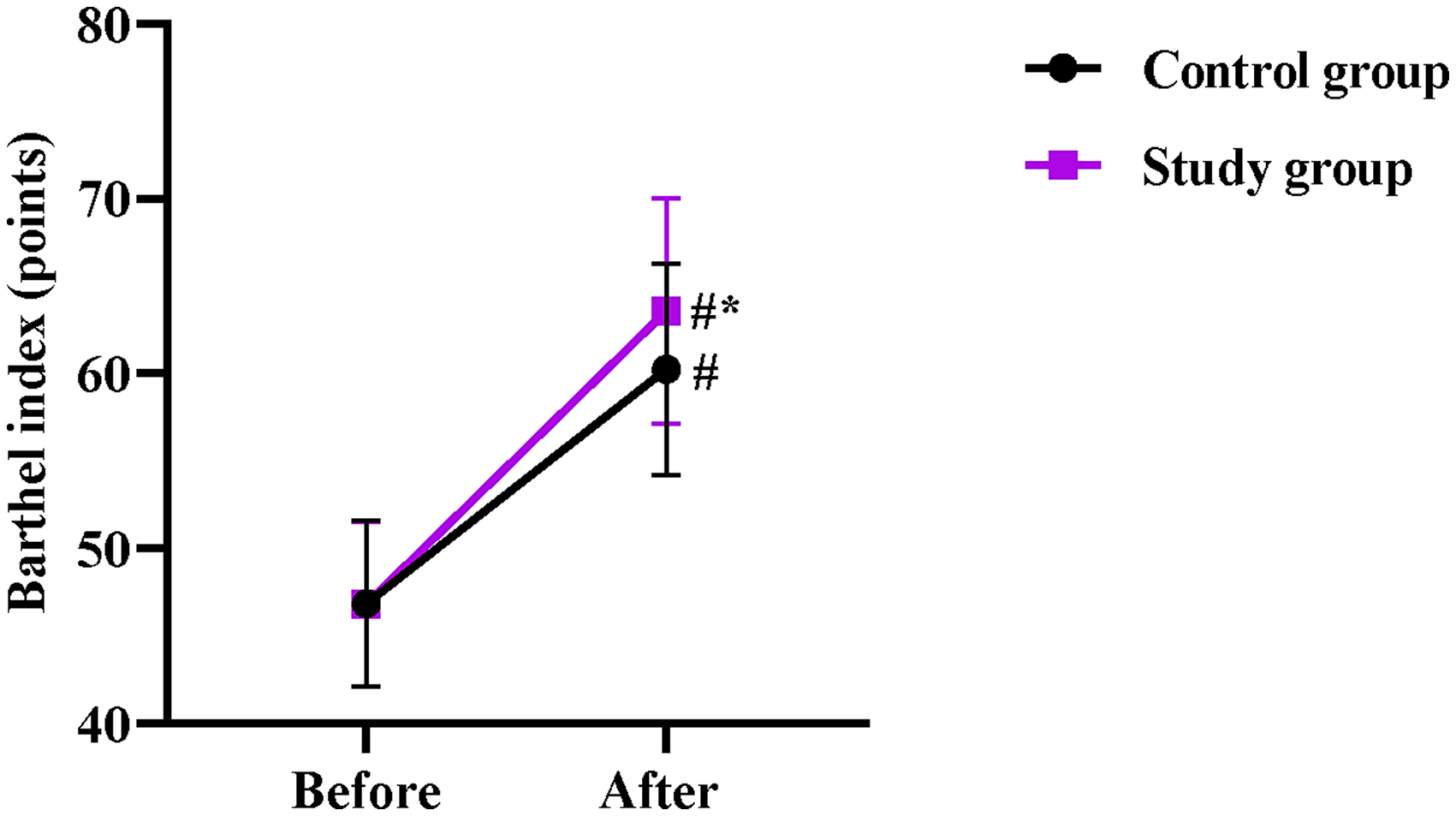
Barthel index scores (mean ± SD) of patients in two groups before and after nursing. ^#^ *p* < 0.05 vs. before nursing within the same group; *p* < 0.05 vs. the control group after nursing.

Before nursing, there was no significant difference in the Barthel index between the two groups (46.85 ± 4.72 vs. 46.82 ± 4.68; mean difference −0.03, 95% CI -1.93 to 1.87; *p* > 0.05). After nursing, Barthel index scores increased in both groups, with higher post-nursing values in the study group than in the control group (63.58 ± 6.45 vs. 60.25 ± 6.05; mean difference 3.33, 95% CI 0.80 to 5.86; Cohen’s d = 0.53; *p* = 0.011) (Figure 5; Supplementary Table 2).

### Incidence of complications in two groups

As shown in Table 1, postoperative complications occurred in 7 of 48 patients (14.58%) in the control group and in 1 of 48 (2.08%) in the study group, corresponding to an absolute risk reduction of 12.50 percentage points (95% CI 1.7–23.3 percentage points; χ^2^ = 4.91; *p* = 0.027).

**Table 1:** Incidence of complications in two groups.

Groups	Cases	Electrode displacement, *n* (%)	Wound infection, *n* (%)	Hypokalemia, *n* (%)	Any complication, *n* (%)
Control group	2 (4.2)	3 (6.3)	2 (4.2)	7 (14.6)	2 (4.2)
Study group	0 (0.0)	1 (2.1)	0 (0.0)	1 (2.1)	0 (0.0)
χ^2^					4.909
*P*					0.027

### Nursing satisfaction in two groups

As shown in Table 2, overall nursing satisfaction (very satisfied or satisfied) was 40 of 48 patients (83.33%) in the control group and 47 of 48 (97.92%) in the study group, yielding an absolute increase in satisfaction of 14.58 percentage points (95% CI 3.3–25.9 percentage points; χ^2^ = 6.01; *p* = 0.014).

**Table 2:** Nursing satisfaction in two groups.

Groups	Cases	Very satisfied, *n* (%)	Satisfied, *n* (%)	Dissatisfied, *n* (%)	Overall satisfaction, *n* (%)
Control group	48	20 (41.7)	20 (41.7)	8 (16.7)	40 (83.3)
Study group	48	26 (54.2)	21 (43.8)	1 (2.1)	47 (97.9)
χ^2^					6.008
*P*					0.014

## Discussion

PD is a chronic developmental motor disorder. Although its pathogenesis is still unclear, the primary pathological changes are characterized by dopamine deficiency due to the degeneration of nerve substantia nigra cells, resulting in a large reduction of dopamine levels in the striatum and the dysfunction of basal ganglia motor circuits (14). With the increase in drug tolerance and adverse reactions, medical treatment for PD often becomes ineffective and needs to be combined with surgical treatment (15). Since the early 1990s, DBS has become an effective surgical treatment for PD (16). In this way, stimulation electrodes can be implanted into specific brain targets in patients through the stereotaxic method. High-frequency electrical stimulation in then applied to alter the excitability of nuclear clusters, control seizures, and improve the clinical symptoms of PD patients (17). However, there are opportunities and risks always associate with this procedure because DBS requires advanced equipment, high technical proficiency, long surgical durations, is expensive, and has limited battery life. These factors have hindered the widespread development of such surgical interventions (18). At the same time, the surgery may lead to complications such as electrode displacement, puncture bleeding, intracranial infection, and permanent nervous system damage (19). To ensure the effectiveness of surgical treatment, it is essential to provide patients with high-quality and careful nursing services in the perioperative period (20).

Routine nursing interventions focus on the patient’s body state, ignoring their individual pathological and physiological needs, and the nursing content is mostly implemented based on experience rather than solid theoretical support, making it increasingly inadequate to adapt to clinical practice (21). 6S management is a new management mode, which is an upgrade version of the previous 5S management mode (22). In this specific operation, the six organic components are interconnected and integrated, rather than functioning independently (23). Within this framework, each component builds on the previous one and prepares the groundwork for the next, forming a continuous management cycle: earlier steps such as sorting, set-in-order, and cleaning create a structured work environment, whereas subsequent steps of standardization, discipline, and safety management further refine and regulate these processes, making nursing work more detailed, standardized, and targeted. For nursing management, this implies that 6S refined individualized nursing can be used as a practical framework to standardize perioperative workflows, optimize the allocation of nursing human and material resources, and strengthen quality control in order to achieve the improvements in efficiency, safety, and patient outcomes observed in our study (24).

In our setting, many elements of the 6S package (such as patient education, early mobilization, pain control, and pressure sore prevention) overlap with what would be considered good standard perioperative care for DBS in high-performing centers; however, the added value of 6S lies in systematically integrating and standardizing these measures within a structured management framework and combining them with individualized nursing plans based on comprehensive patient assessment, which together may explain the more consistent and pronounced improvements observed in the study group.

In addition to the conceptual framework of 6S, our findings are also consistent with emerging evidence that structured, protocol-driven perioperative care can improve recovery after DBS and other neurosurgical procedures. Enhanced recovery after surgery (ERAS) principles have increasingly been applied in neurosurgery and elective craniotomy, where multimodal, pathway-based interventions have been reported to reduce complications and shorten hospital stay without compromising safety (25). In the context of DBS, fast-track postoperative protocols that standardize monitoring, pain control, and early mobilization have been described as safely avoiding routine ICU admission and facilitating early transfer to the ward and discharge (26, 27). Our 6S refined individualized nursing model similarly emphasizes standardized perioperative workflows, intensified nursing surveillance, and early mobilization within a structured framework, and the observed reductions in time to ambulation and postoperative LOS in the study group align with these enhanced-recovery concepts.

In addition, the individualized component of our 6S approach fits within a broader literature on specialist PD nursing and personalized care models. Trials and economic evaluations of community-based PD nurse specialists have suggested that, although effects on motor outcomes may be modest or inconsistent, specialist nursing can improve patients’ sense of wellbeing and quality of life without increasing overall healthcare costs (28–30). Recent narrative reviews and meta-analyses of Parkinson nurses similarly emphasize their role in coordinating multidisciplinary care, providing tailored education, and addressing non-motor symptoms and psychosocial needs (31, 32). Our results extend these observations into the perioperative DBS setting by showing that a structured, 6S-based nursing framework combined with individualized care plans is associated with lower anxiety and depression scores, better functional recovery, and higher nursing satisfaction during the acute hospitalization period in PD patients undergoing DBS.

Our findings indicate that the SG had significantly shorter operation time, time to first ambulation, and postoperative hospital stay compared to the CG, and the VAS score in the SG presented lower when compared with the CG at 6 h, 12 h, 24 h along with 48 h after surgery, suggesting that the application of 6S refined individualized nursing management could promote postoperative recovery along with reduce the degree of pain in the perioperative period of PD patients undergoing DBS, which was in line with previous studies (33).

Depressive disturbances are common in PD patients and impact many other clinical aspects of this disease (34). In our study, we proved that after nursing, the SAS and SDS scores in the SG presented reduction when comparing with the CG, and the POMA score and Barthel index in the SG presented higher when comparing with the CG, implying that the application of 6S refined individualized nursing management could reduce anxiety and depression, promote the balance ability and daily living ability in the perioperative period of PD patients undergoing DBS, which was similar to previous report (28).

In addition, our study indicated that in comparison with the CG, the incidence of complications in the SG presented a reduction, and the nursing satisfaction of patients in the SG presented higher, suggesting that the application of 6S refined individualized nursing management could reduce the occurrence of complications along with promote nursing satisfaction in the perioperative period of PD patients undergoing DBS.

This study has several limitations. First, it was a single-center retrospective observational study without randomized allocation, so residual confounding cannot be ruled out. Second, only age and sex could be systematically compared between groups; key PD- and DBS-related baseline variables such as disease duration, Hoehn-Yahr stage, UPDRS/MDS-UPDRS scores, DBS target and laterality, cognitive and psychiatric status, comorbidities, fall risk, and antiparkinsonian medication burden were not uniformly available in the electronic database and thus were not analyzed. Consequently, although the observed between-group differences were statistically significant, the possibility that unmeasured baseline imbalances contributed to these differences cannot be excluded, and the results should be interpreted as exploratory and hypothesis-generating. Third, the SAS, SDS, POMA, and the Barthel index were assessed only in the short-term perioperative period (within 24 h before surgery and within 24 h before discharge), and nursing satisfaction was measured once at discharge; longer-term fluctuations in mood, function, and satisfaction after DBS programming and medication adjustments were not captured. Fourth, nursing satisfaction was evaluated using a hospital-designed nursing evaluation scale that had not undergone formal psychometric validation in PD or DBS populations, which may affect the precision and generalizability of the satisfaction findings. Fifth, only a limited set of nursing-recorded perioperative complications (electrode displacement, wound infection, and hypokalemia) was analyzed; other important DBS-related adverse events were not systematically and uniformly collected and therefore were not included, which restricts the comprehensiveness of the safety assessment. Moreover, we did not adjust for multiple comparisons across the several outcomes analyzed, and we relied on univariable comparisons without multivariable regression to adjust for potential confounders. Together, these factors further limit the robustness of causal inference, and the findings should be regarded as exploratory and hypothesis-generating. Future multicenter prospective randomized studies with comprehensive baseline characterization, predefined primary outcomes, formal sample-size calculations, and standardized fidelity and safety assessments are needed to confirm our findings. Furthermore, although the 6S refined individualized nursing model was implemented as part of routine quality improvement activities and did not require dedicated external funding, we did not perform a formal evaluation of implementation costs, training time, or nursing workload per patient. Future studies should incorporate cost-effectiveness and workload analyses to better assess the feasibility and scalability of this model across different healthcare settings.

## Conclusion

In this single-center retrospective cohort, 6S refined individualized nursing management was associated with faster postoperative recovery, lower anxiety and depression scores, better balance ability and activities of daily living, fewer nursing-recorded perioperative complications, and higher nursing satisfaction among PD patients undergoing DBS. However, in view of the retrospective design, limited baseline characterization, absence of multivariable adjustments, and lack of formal control for multiple comparisons, these associations should be interpreted as exploratory and hypothesis-generating, and they need to be validated in larger multicenter prospective randomized studies.

## Data Availability

The original contributions presented in the study are included in the article/Supplementary material, further inquiries can be directed to the corresponding authors.
