# Supplementary material for: Effect of 6S refined individualized nursing management in the perioperative period of Parkinson’s disease patients undergoing deep brain stimulation
Source: Front Neurol. 2026 Jun 3;17:1671449. doi: 10.3389/fneur.2026.1671449 (PMC13271964; doi:10.3389/fneur.2026.1671449)
Supplement: Supplementary file 1 [file Table_1.DOCX]

Supplementary Table 1 Comparison of routine perioperative nursing and 6S refined individualized nursing management

| **Domain** | **Routine nursing (control group)** | **6S refined individualized nursing (study group)** |
| --- | --- | --- |
| Preoperative assessment and education | Basic preoperative assessment of general condition and neurological status; provision of general information about DBS surgery, perioperative precautions, and medication instructions. | Structured preoperative assessment including age, disease duration and main symptoms, comorbidities, functional status, cognitive and emotional state, and family support; tailored health education focusing on individual concerns, expected benefits and risks of DBS, perioperative cooperation, and home care, with active involvement of family members. |
| Environmental and organizational management | General ward organization and routine cleaning according to departmental practice. | Application of 6S principles (Sort, Set in order, Shine, Standardize, Sustain, Safety) to reorganize ward layout and materials: DBS-related devices and supplies sorted, labeled, and stored in designated locations; visual management of medications and wound-care materials; regular inspection and documentation of cleanliness and safety; development of standardized operating procedures and visual flowcharts for key perioperative nursing steps. |
| Postoperative monitoring | Routine monitoring of vital signs and neurological status; observation of consciousness, wound condition, and limb movement according to standard ward practice. | Intensified and protocolized monitoring based on 6S workflows: structured observation of vital signs, neurological status, electrode and wound condition, and limb function; standardized documentation forms and time points; early identification and targeted management of abnormal findings according to predefined procedures. |
| Early mobilization | Encouragement of early ambulation after surgery according to general clinical experience and physician orders. | Individualized mobilization plan based on preoperative balance and ADL status, intraoperative factors, and postoperative recovery; graded mobilization targets and timelines; close supervision during initial mobilization to prevent falls; dynamic adjustment of the plan according to daily performance. |
| Pain management | Conventional pharmacological analgesia prescribed by physicians; general advice on pain reporting. | Combined pharmacological and non-pharmacological pain management based on individual pain assessment: regular VAS evaluation, timely adjustment of analgesic regimen, and use of non-drug measures (e.g., positioning, relaxation techniques) integrated into daily care; enhanced communication to explore and address pain-related anxiety. |
| Complication prevention (falls, pressure sores, etc.) | General measures for prevention of falls and pressure sores (e.g., bed rails, regular turning, basic skin care) according to routine practice. | Risk stratification for falls and pressure sores based on comprehensive assessment; targeted preventive measures (e.g., individualized turning schedule, optimized positioning, protective devices, enhanced environmental safety checks); specific education for patients and caregivers on electrode and wound protection and fall prevention. |
| Psychological support | General psychological support and reassurance provided during routine interactions. | Targeted psychological nursing based on SAS/SDS and clinical evaluation: identification of high-risk patients for anxiety and depression, provision of structured counseling, emotional support, and coping strategies; frequent communication with patients and families to address specific fears related to DBS surgery and recovery. |
| Discharge planning and follow-up guidance | Routine discharge instructions regarding medication, wound care, and outpatient follow-up. | Enhanced discharge planning with individualized written and verbal guidance on medication management, wound and electrode protection, activity progression, home safety, psychological adjustment, and timing of follow-up visits; confirmation of patient and caregiver understanding. |
| Implementation oversight | General supervision by the charge nurse according to routine ward management. | Continuous implementation oversight by the head nurse and senior nurses through regular ward rounds, review of individualized nursing plans and records, and feedback during shift handovers; identification and correction of deviations from 6S procedures. |
