# Supplementary material for: Effect of 6S refined individualized nursing management in the perioperative period of Parkinson’s disease patients undergoing deep brain stimulation
Source: Front Neurol. 2026 Jun 3;17:1671449. doi: 10.3389/fneur.2026.1671449 (PMC13271964; doi:10.3389/fneur.2026.1671449)
Supplement: Supplementary file 2 [file Table_2.DOCX]

Supplementary Table 2 Comparison of perioperative, psychological, pain, balance, and functional outcomes between the two groups

| **Outcome / time point** | **Control group (n=48), mean±SD** | **Study group (n=48), mean±SD** | **Mean difference (study - control) (95% CI)** | **Cohen’s d** | **P value** |
| --- | --- | --- | --- | --- | --- |
| Operation time (h) | 3.19±0.32 | 2.83±0.37 | -0.36 (95% CI -0.50 to -0.22) | 1.05 | <0.001 |
| Time to first getting out of bed (h) | 14.89±2.74 | 13.41±1.51 | -1.48 (95% CI -2.38 to -0.58) | 0.67 | 0.002 |
| Postoperative hospitalization time (d) | 11.22±0.95 | 10.34±0.71 | -0.88 (95% CI -1.22 to -0.54) | 1.05 | <0.001 |
| SAS score, before nursing | 61.65±6.17 | 61.68±6.21 | 0.03 (95% CI -2.48 to 2.54) | – | >0.05 |
| SAS score, after nursing | 30.25±3.06 | 21.52±2.15 | -8.73 (95% CI -9.80 to -7.66) | 3.30 | <0.001 |
| SDS score, before nursing | 60.12±6.05 | 60.18±6.13 | 0.06 (95% CI -2.41 to 2.53) | – | >0.05 |
| SDS score, after nursing | 30.26±3.05 | 20.25±2.04 | -10.01 (95% CI -11.06 to -8.96) | 3.86 | <0.001 |
| VAS score, 6 h after surgery | 2.68±0.27 | 2.06±0.21 | -0.62 (95% CI -0.72 to -0.52) | 2.56 | <0.001 |
| VAS score, 12 h after surgery | 2.35±0.24 | 1.62±0.17 | -0.73 (95% CI -0.81 to -0.65) | 3.51 | <0.001 |
| VAS score, 24 h after surgery | 2.05±0.21 | 1.21±0.12 | -0.84 (95% CI -0.91 to -0.77) | 4.91 | <0.001 |
| VAS score, 48 h after surgery | 1.48±0.15 | 0.82±0.08 | -0.66 (95% CI -0.71 to -0.61) | 5.49 | <0.001 |
| POMA score, before nursing | 10.68±1.07 | 10.65±1.05 | -0.03 (95% CI -0.46 to 0.40) | – | >0.05 |
| POMA score, after nursing | 15.26±1.53 | 17.42±1.75 | 2.16 (95% CI 1.49 to 2.83) | 1.31 | <0.001 |
| Barthel index, before nursing | 46.85±4.72 | 46.82±4.68 | -0.03 (95% CI -1.93 to 1.87) | – | >0.05 |
| Barthel index, after nursing | 60.25±6.05 | 63.58±6.45 | 3.33 (95% CI 0.80 to 5.86) | 0.53 | 0.011 |

Note: Data are presented as mean ± standard deviation (mean±SD). Between-group comparisons were performed using independent-samples t-tests. Effect size was quantified using Cohen’s d. SAS, Self-Rating Anxiety Scale; SDS, Self-Rating Depression Scale; VAS, Visual Analog Scale for pain; POMA, Performance-Oriented Mobility Assessment; ADL, activities of daily living (assessed by the Barthel index); 95% CI, 95% confidence interval.
